# Comprehensive Integrated Single-Cell Whole Transcriptome Analysis Revealed the p-EMT Tumor Cells—CAFs Communication in Oral Squamous Cell Carcinoma

**DOI:** 10.3390/ijms23126470

**Published:** 2022-06-09

**Authors:** Nam Cong-Nhat Huynh, Tze-Ta Huang, Chi Thi-Kim Nguyen, Fang-Kuei Lin

**Affiliations:** 1 Laboratory of Oral-Maxillofacial Biology, Faculty of Odonto-Stomatology, University of Medicine and Pharmacy at Ho Chi Minh City, Ho Chi Minh City 749000, Vietnam; 2 Institute of Basic Medical Sciences, College of Medicine, National Cheng Kung University, Tainan 701401, Taiwan; 3 Institute of Oral Medicine, College of Medicine, National Cheng Kung University, Tainan 701401, Taiwan; meitung0703@gmail.com; 4 Department of Dentistry, College of Medicine, National Cheng Kung University, Tainan 701401, Taiwan; 5 Department of Oral Pathology, Faculty of Odonto-Stomatology, University of Medicine and Pharmacy at Ho Chi Minh City, Ho Chi Minh City 749000, Vietnam; drnguyenchifos@ump.edu.vn

**Keywords:** single-cell RNA sequencing, oral squamous cell carcinoma, cancer-associated fibroblasts, partial epithelial–mesenchymal transition, metastasis

## Abstract

Cancer-associated fibroblasts (CAFs) and partial epithelial–mesenchymal transition (p-EMT) tumor cells are closed together and contribute to the tumor progression of oral squamous cell carcinoma (OSCC). In the present study, we deeply analyzed and integrated OSCC single-cell RNA sequencing datasets to define OSCC CAFs and p-EMT subpopulations. We highlighted the cell–cell interaction network of CAFs and p-EMT tumor cells and suggested biomarkers for the diagnosis and prognosis of OSCC during the metastasis condition. The analysis discovered four subtypes of CAFs: one p-EMT tumor cell population, and cycling tumor cells as well as TNFSF12-TNFRSF25/TNFRSF12A interactions between CAFs and p-EMT tumor cells during tumor metastasis. This suggests the prediction of therapeutically targetable checkpoint receptor–ligand interactions between CAFs and p-EMT tumor cells in OSCC regarding the metastasis status.

## 1. Introduction

Head and neck cancers, including head and neck squamous cell carcinoma (HNSCC) and oral squamous cell carcinoma (OSCC), are a heterogeneous collection of malignancies of the upper aerodigestive tract, salivary glands, and thyroid [1,2]. It is estimated that more than 830,000 individuals have suffered from this disease, which caused over 430,000 deaths worldwide each year [3]. Molecular, cellular, and tissue-level interactions facilitate critical transitions in cancer [4]; however, a perspective on changes in oral tumor ecosystems has not been investigated thoroughly. This leads to difficulties in prognosis and in the development of risk-stratified cancer screening, precision screening, and treatment strategies. Intra-tumoral heterogeneity represents a major challenge in oncology [5]. Among emerging technologies, single-cell RNA sequencing (scRNA-seq) has facilitated the identification of developmental hierarchies, drug resistance programs, and patterns of immune infiltration relevant to tumor biology, diagnosis, and therapy [6].

Tumor microenvironment (TME) is considered a multicellular ecosystem interacting closely with tumor cells, contributing to tumorigenesis. It contains mesenchymal, endothelial, and hematopoietic original cells arranged in the extracellular matrix (ECM) and contributes to the initiation and progression of cancers. Cancer-associated fibroblasts (CAFs) are the most dominant components in the tumor stroma. CAFs help to generate and remodel the ECM structure within TME [7]. Because of the complicated heterogeneity, CAFs’ origin, subtypes, and biology remain unclear [8]. During the progression of epithelial cancers, partial epithelial–mesenchymal transition (p-EMT) is the intermediate state of epithelial–mesenchymal transitions (EMTs), when the cancer cells exhibit both mesenchymal and epithelial signatures [9]. p-EMT plays a vital role during the invasion and metastasis processes in many types of epithelial cancers. In fact, CAFs and p-EMT tumor cells are closed together and contribute to tumor progression, including HNSCC and OSCC [10]. However, the underlying mechanisms of this bond remain vague [11].

A previous study has reported immune and non-immune cell interactions at a single-cell level within the tumor microenvironment of HNSCC [12]. Interactions between CD8+ T cells and B CD45–endothelial cells, epithelial cells, fibroblasts, and pericytes or myeloid antigen-presenting cells, especially tumor-associated macrophages, were found to be the dominant contributors to PD-L1 and other immune checkpoint ligands. However, there was no investigation into the communication of closed tumor cells, including CAFs and p-EMT tumor cells in OSCC. In the present study, we deeply analyzed, integrated, and published our own OSCC sc-RNAseq data to define OSCC CAFs and p-EMT subpopulations. We highlighted the cell–cell interaction network of CAFs and p-EMT tumor cells and suggested biomarkers for the diagnosis and prognosis of OSCC during the metastasis condition. This study helps answer fundamental questions regarding aspects of the biological nature of the OSCC tumor microenvironment and serves as a guide to unraveling the nature of OSCC.

## 2. Results

### 2.1. Integrated Single-Cell RNA Sequencing Mapped OSCC Microenvironment Cell Types across the Metastasis Condition

**Figure 1:**
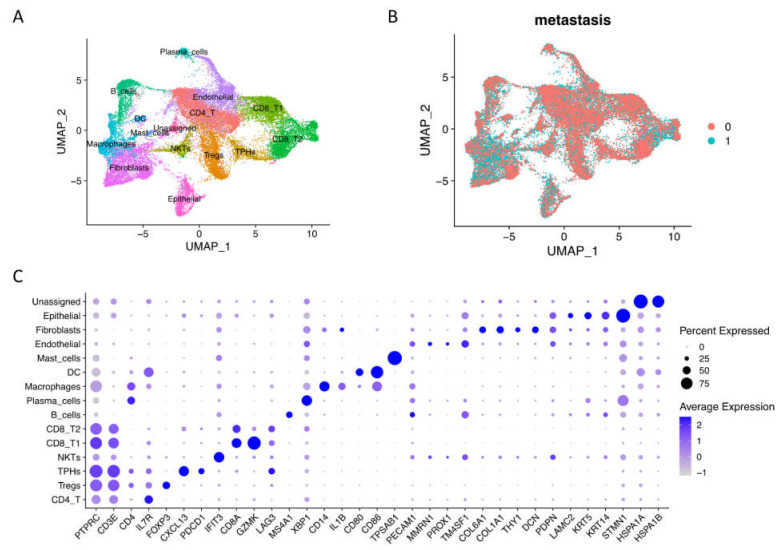
Single-cell RNA-seq integration of oral squamous cell carcinoma data. (**A**) Integrated cells were visualized by UMAP with 15 main cell types assigned names based on canonical markers. (**B**) Integrated scRNA-seq data included 16,817 non-lymph node metastasis cells (0, N0) and 13,183 lymph node metastasis cells (1, N1 to Nx). (**C**) Dotplot of the average expression of the main markers of each cell type in oral squamous cell carcinoma integration data.

To explore the big spectrum of oral tumor microenvironment under nonmetastasis and metastasis conditions, we integrated GSE103322 and GSE164690 datasets, and our own published data resulted in 60,662 raw cells (oral cavity site, primary tumor tissues, and HPV with metastasis information) with 28,135 genes from 31 patients. After quality control, we obtained 42,499 cells and used 30,000 resampling cells for visualization, including 16,817 non-lymph node metastasis cells and 13,183 lymph node metastasis cells (Figure 1A,B). After clustering, we identified 15 main cell types by canonical marker expression (Figure 1C, Supplementary Materials File S1). Differential markers named T cells (PTPRC/CD45+ and CD3E+) include conventional CD4 T cells (IL7R+), regulatory CD4 Tregs (FOXP3+), nature killer T cells (high interferon signaling), conventional CD8 T cells (GZMK+), and exhausted CD8 T cells (LAG3+ and TIM3+). Interestingly, we found the existence of peripheral helper T cells (Tph) with CXCL13+ and PDCD1/PD-1+ expressions [13]. Tumor immune cells (PTPRC/CD45+) also consisted of B cells (MS4A1/CD20+), plasma cells (XBP1+), macrophages (CD14+), dendritic cells (CD80+, CD86+), and mast cells (TPSAB1+). Stromal cells (or cancer-associated cells) included endothelial cells (PECAM1/CD31+ and MMRN1+), fibroblasts (COL6A1+, COL1A1+, THY1+, and DCN+), epithelial cells (LAMC2+, KRT5+, and KRT14+), and unassigned name cells (high heat shock proteins expression), suggesting that they were cancerous cells. Moreover, pseudo-bulk RNA-seq analysis revealed the different gene expressions of each cell type in non- vs. metastasis conditions (Supplementary Materials Figure S1A). Most genes upregulated in non-metastasis conditions were related to immune responses and cellular functions, while upregulated genes in metastasis conditions were related to cancer genes and invasion, especially in endothelial cells, fibroblasts, and epithelial cells (Supplementary Materials File S2).

### 2.2. Cellular Community of OSCC Fibroblasts and Epithelial Cells at Single-Cell Level

**Figure 2:**
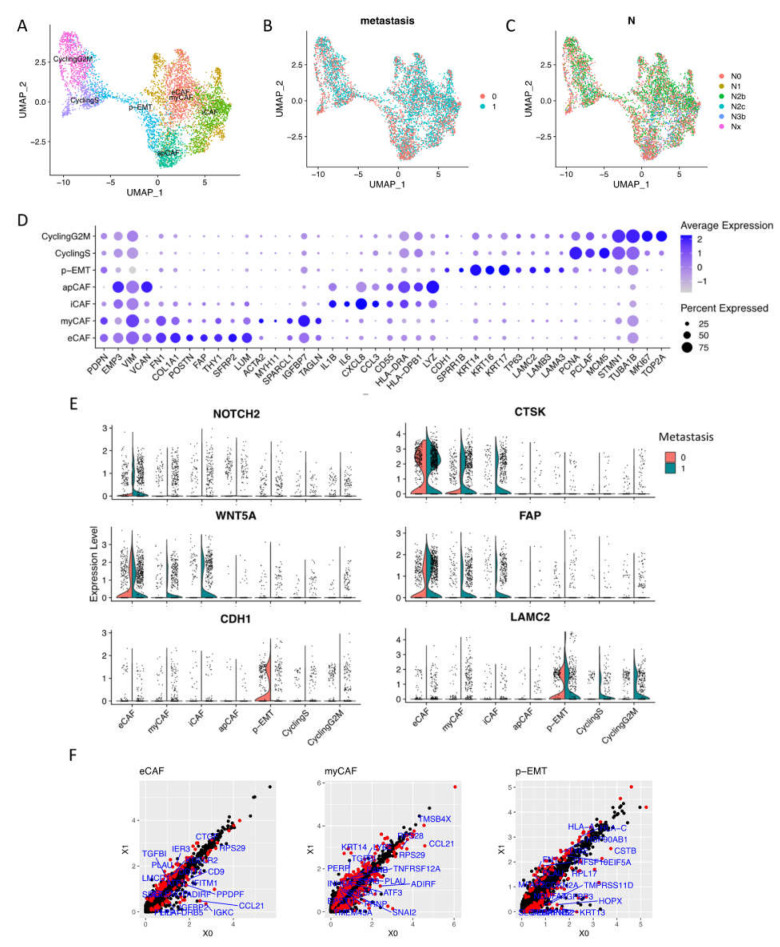
Sub-clusters of fibroblasts and epithelial cells. (**A**) Cells were sub-clustered into 7 sub-types and visualized by UMAP. (**B**,**C**) Integrated data included 2,270 non-lymph node metastasis cells (0, N0) and 3,239 lymph node metastasis cells (1, N1 to Nx). (**D**) Dotplot of average expression of the main markers of each sub-type in integration data. (**E**) Expressions of distinguished markers in non- (0) vs. metastasis (1) OSCC. (**F**) Differentiation of gene expression of eCAFs, myCAFs, and p-EMT tumor cells by pseudo bulk RNA-seq analysis in non- (X0) vs. metastasis (X1) conditions. Red dots are significantly different genes; black dots are total genes; blue labels are the top 20 significant different genes by adjusted *p* value.

Focusing on CAFs and epithelial cells, we performed sub-clustering to zoom in on the cellular component of these cell types (Figure 2A,D, Supplementary Materials File S1). These integrated data included 2270 non-lymph node metastasis conditions (0, N0) and 3,239 lymph node metastasis conditions (1, N1 to Nx) of fibroblasts and epithelial cells Figure 2B,C).

CAF clusters generally expressed tumorigenesis EMP3 and EMT-related VIM (Vimentin) genes. A high-resolution analysis revealed four subtypes of CAFs that upregulated elastic fibers and ECM restructuring gene expressions (we named as eCAFs) such as COL1A1, POSTN (Periostin), FAP (Fibroblast Activation Protein Alpha), THY1 (CD90), FN1 (Fibronectin), and FBLN1 (Fibulin-1). eCAFs also strongly expressed cancer-associated genes including tumorigenesis SFRP2 (Secreted frizzled-related protein 2) and proteoglycan LUM (Lumican). We also identified myofibroblastic CAFs as previous reports (myCAFs) that expressed ACTA2 (α-SMA), MYH11, and TAGLN (Transgelin) [14,15]. Inflammatory CAFs (iCAFs), which expressed low levels of α- but high levels of chemokines and cytokines (IL1B, IL6, CXCL8, and CCL3), were confirmed in OSCC integrated single-cell data. Finally, we found antigen-presenting CAFs (apCAFs) that expressed MHCII genes.

For epithelial lineage, proliferating cells were dominant, including one cell cycling sub-cluster at phase G1/S (DNA repair genes—PCNA and PCLAF—and cell cycle regulation gene—MCM5) and one cell cycling sub-cluster at phase G2/M (cell division genes STMN1 and TUBA1B, and cell proliferation genes MKI67 and TOP2A). Notably, we found p-EMT tumor cells (p-EMT sub-cluster) that highly expressed CDH1 (E-cadherin), keratin KRT14/16/17, TP63 (Tumor protein p63), and basement membrane ECM glycoprotein- Laminin family (LAMC2, LAMB3, LAMA3…).

From non- to metastasis OSCC, the expressions of well-known distinguished markers such as NOTCH2, WNT5A, FAP, and CTSK (Cathepsin K) were upregulated in metastasis CAFs. LAMC2 was upregulated in metastasis epithelial cells, while CDH1 (E-cadherin) was faded in metastasis p-EMT tumor cells (Figure 2E). The differentiation of gene expression of eCAFs, myCAFs, and p-EMT tumor cells by pseudo-bulk RNA-seq analysis in non- vs. metastasis conditions was presented in Figure 2F. For iCAFs, apCAFs, CyclingS, and CyclingG2M, we can find the different genes in Supplementary Materials Figure S1B. Enrichment analysis highlighted the difference in GO terms between non- vs. metastasis conditions (Supplementary Materials Figure S2); for instance, metastasis eCAFs increased response to cytokine and ECM organization. Metastasis myCAFs increased apoptosis, response to cytokine, and collagen formation. On the other hand, non-metastasis iCAFs and apCAFs increased the PD-1 signaling pathway and adaptive immune response. Significantly, metastasis p-EMT cells upregulated the positive regulation of cell motility, suggesting the invasion process. Gene–disease association analysis emphasized oral cavity carcinoma, malignant neoplasm of mouth/head and neck, carcinomatosis, and lymphatic metastasis signatures in metastasis sub-clusters, especially in eCAFs, myCAFs, p-EMT, and cycling epithelial tumor cells.

### 2.3. Cell–Cell Interaction of OSCC CAFs and p-EMT Tumor Cells in Non-Metastatsis and Metastasis Conditions

**Figure 3:**
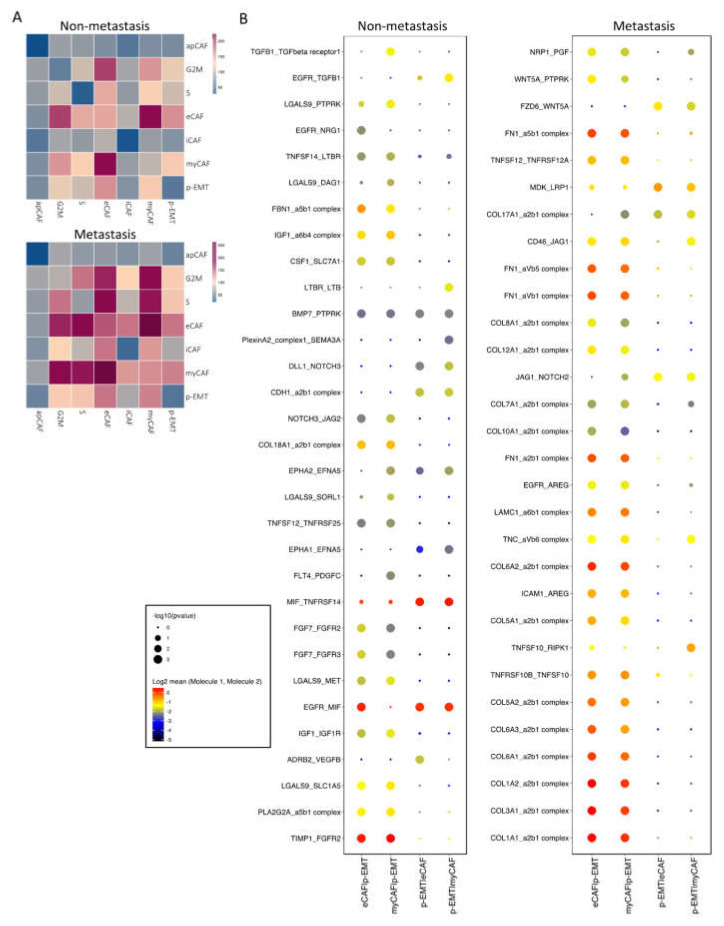
Cell–cell communication analysis in sub-clusters of fibroblasts and epithelial cells. (**A**) Heatmap of all interactions in non- vs. metastasis conditions by CellPhoneDB analysis. (**B**) Top exclusive 30 significant interactions upregulated only in non- or metastasis conditions between p-EMT cluster and eCAF/myCAF clusters (dot’s sizes illustrate the -log10(pvalue), in which the bigger size presents the lower *p* value; dot’s colors illustrate the mean interaction number, in which the redder color presents the higher number of mean interactions between 2 clusters.

**Figure 4:**
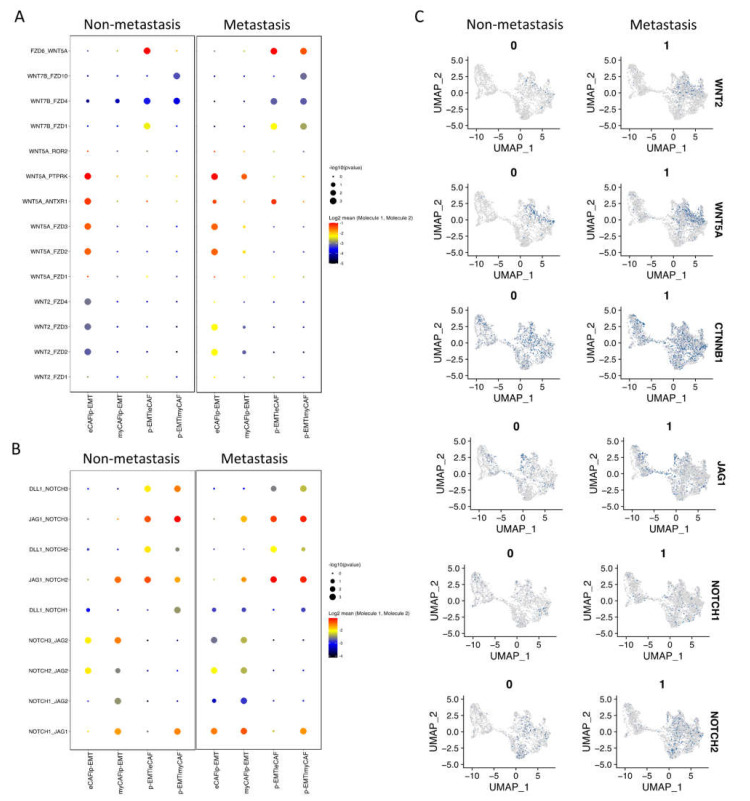
WNT and NOTCH signaling pathways increased in metastasis p-EMT cluster and eCAF/myCAF clusters. (**A**) Significant WNT signaling pathway communications upregulated in metastasis conditions between p-EMT cluster and eCAF/myCAF clusters (with compared non-metastasis condition). (**B**) Significant NOTCH signaling pathway communications upregulated in metastasis condition between p-EMT cluster and eCAF/myCAF clusters (with compared non-metastasis condition). (**C**) WNT and NOTCH signaling pathway-related gene expressions in integrated single-cell RNA-seq of fibroblasts and epithelial cells in non- vs. metastasis conditions.

Next, we investigated the interaction between CAFs and epithelial cell clusters at the single-cell level; the CellPhoneDB program counted a total of 1110 interactions in non-metastasis clusters and 1181 interactions in metastasis clusters. There were 382 and 350 significant interactions in non- and metastasis clusters, respectively (Supplementary Materials File S3). In non-metastasis clusters, the highest significant communication number was between eCAFs and myCAFs (Figure 3A), which can be explained by connective tissue interactions via the ECM network (Supplementary Materials File S3). In metastasis clusters, the number of significant interactions increased dramatically, especially between eCAFs, myCAFs, and other clusters (Figure 3A). Considering the immune functions of iCAFs and apCAFs, as well as the gene signatures of cell cycling epithelial tumor cells, we focused on the cell–cell communication of eCAFs/myCAFs and p-EMT to investigate what was different during the EMT process of cells in non- vs. metastasis conditions. Figure 3B presented the top 30 exclusively upregulated interactions in non- vs. metastasis conditions between eCAFs/myCAFs and p-EMT. We found that, in non-metastasis conditions, there was a high number of interactions for cell signaling, cell differentiation, cell–cell adhesion, ECM composition, and wound healing such as FN1 (Fibronectin)—a5b1 complex (Integrin); CDH1—a2b1 complex; TIMP1 (matrix metalloproteinases—MMPs inhibitor)—FGFR2 (Fibroblast growth factor receptor 2); and EGFR-TGFB1. However, in metastasis conditions, the numbers of integrin and collagen complex interactions between eCAFs/myCAFs and p-EMT increased notably including COL1A1/1A2/3A1/5A1/5A2/6A1/6A2/6A3/7A1/8A1/10A1/12A1/17A1—a2b1 complex; LAMC1—a6b1 complex; and FN1—aVb1/aVb5/a5b1 complex. We also noted the escalation of cancer-associated WNT and NOTCH signaling interactions in metastasis conditions between eCAFs/myCAFs and p-EMT (Figure 3B and Figure 4A,B), such as WNT2/5A—FZD2/6 interactions and NOTCH1/2/3—JAG1/2 interactions. The upregulation of WNT2, WNT5A, CTNNB1 (β-catenin), JAG1, NOTCH1, and NOTCH2 gene expressions in metastasis conditions confirmed the cell–cell communication analysis between eCAFs/myCAFs and p-EMT (Figure 4C).

### 2.4. The p-EMT Tumor Cells—CAFs Communication Highlighted the Different Roles of TNFRSF25 in Non-Metastasis and TNFRSF12A in Non-Metastasis OSCC

**Figure 5:**
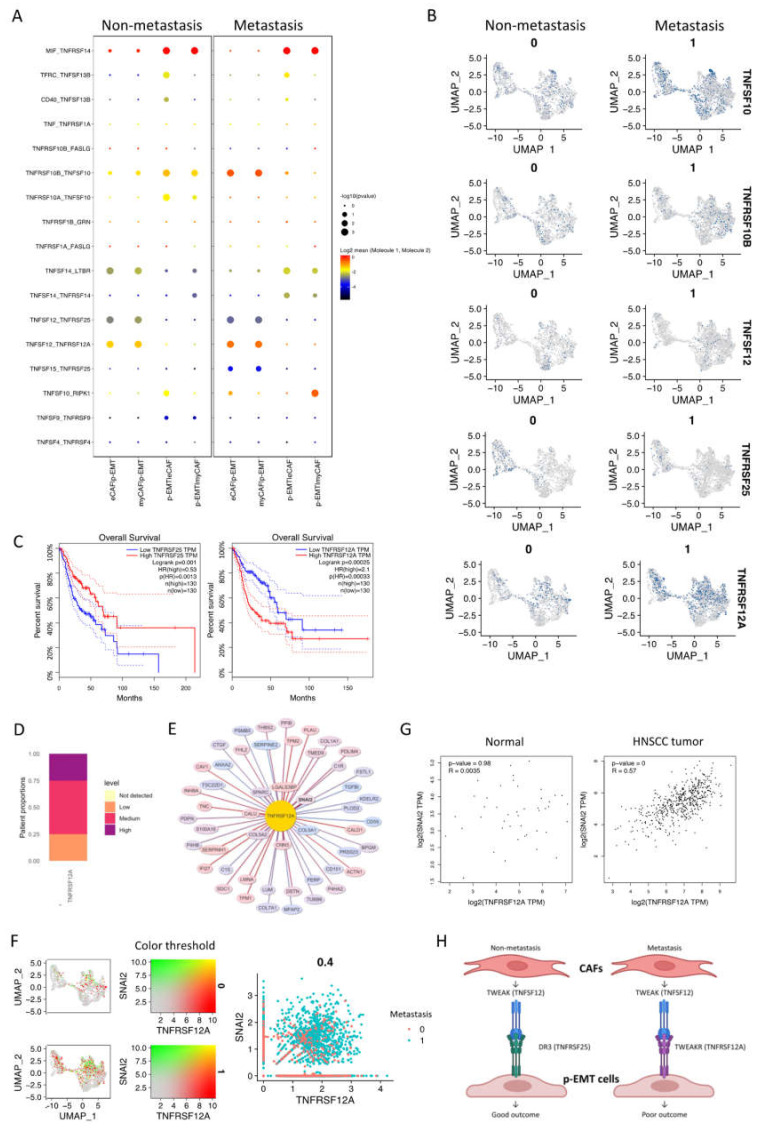
Different roles of TNFRSF25 and TNFRSF12A in non- and metastasis conditions of oral cancer. (**A**) Significant TNF signaling pathway communications were altered in non- vs. metastasis conditions between p-EMT and eCAF/myCAF clusters. (**B**) TNF signaling pathway-related gene expression in integrated single-cell RNA-seq of fibroblasts and epithelial cells in non- vs. metastasis conditions. (**C**) Survival plots of head and neck cancer patients with high TNFRSF25 (left panel) and TNFRSF12A (right panel) expression from TCGA database. (**D**) Proportion of TNFRSF12A expression in head and neck cancer patients from Human Protein Atlas. (**E**) Top 100 TNFRSF12A-correlated genes in integrated single-cell RNA-seq of fibroblasts and epithelial cells. (**F**) Co-expression (left) and scatter (right) plots of TNFRSF12A (red) and SNAI2 (green) in integrated single-cell RNA-seq of fibroblasts and epithelial cells in non- (0) vs. metastasis (1) conditions. (**G**) Correlation plots of TNFRSF12A and SNAI2 gene expressions in HNSCC non-tumor samples (normal cells) and tumor tissues from TCGA database. (**H**) Conceptual diagram (visualized by BiorRender) of p-EMT cells and CAFs communication in non- vs. metastasis oral squamous cell carcinomas via TWEAK (TNFSF12)-DR3 (TNFRSF25)/TWEAKR (TNFRSF12A).

In addition to the upregulation of integrin and collagen complexes, WNT and NOTCH signaling interactions in non- vs. metastasis conditions between eCAFs/myCAFs and p-EMT, we found that that TNF signaling interactions were also increased in metastasis conditions (Figure 3B). We further discovered all significant TGF signaling interactions in Figure 5A. The results highlighted the different patterns of TNFSF10 (TNF-related apoptosis-inducing ligand/TRAIL, an apoptosis-inducing ligand)—TNFRSF10A/B (Death receptor 4/5) between eCAFs/myCAFs and p-EMT. In detail, non-metastasis p-EMT tumor cells received more apoptosis signaling from eCAFs/myCAFs ligands while metastasis from eCAFs/myCAFs received more apoptosis signaling from p-EMT tumor cells via TNFSF10 (Figure 5A,B).

Remarkably, there were converse pairs of interactions via TNFSF12 (TWEAK—an apoptosis-inducing ligand)—TNFRSF25 (Death receptor 3) and TNFSF12—TNFRSF12A (TWEAK receptor/TWEAKR) (Figure 5A,B). TNFRSF12 interacted with both TNFRSF12A and TNFRSF25 in non- and metastasis conditions. However, in non-metastasis conditions, the TNFSF12 ligand from eCAFs/myCAFs increased communications with TNFRSF25, while in metastasis conditions, this ligand increased communication with TNFRSF12A in p-EMT tumor cells. The gene expression of TNFRSF25 and TNFRSF12A confirmed the difference between the downregulation of TNFRSF25 and the upregulation of TNFRSF12A in metastasis epithelial clusters. From the TCGA-HNSCC database, we analyzed the overall survival of 260 HNSCC patients (*n* = 130 for each group). The Kaplan–Meier plots revealed very significant outcomes (Figure 5C). At 25 months, patients with a high expression of TNFRSF25 were observed with 80% cumulative survival probability, compared to 60% of the low expressing group. After 90 months (about 7.5 years), TNFRSF25 high expressing groups maintained a 40% cumulative survival probability up to 200 months. However, the survival probability of the low expressing group was 15% up to 150 months of observation. Surprisingly, patients with high expressions of TNFRSF12A were observed with a 50% cumulative survival probability compared to 80% of the low expressing group at 25 months. After 90 months, these probabilities were 30% and 38%, respectively. Disease-Specific Survival and Progression-Free Interval analyses of TNFRSF25 and TNFRSF12A in HNSCC patients from TCGA database are presented in Supplementary Materials Figure S3A. The results showed similar trends of the opposing roles of TNFRSF25 and TNFRSF12A in HNSCC patients in Overall Survival analysis. In the total number of patients, TNFRSF12A was expressed as 25% at low, 50% at medium, and 25% at the high level (Figure 5D). The protein expression of TWEAKR by immunohistochemistry confirmed low, medium, and high expressions in HNSCC patients’ tumors (Supplementary Materials Figure S3B). Correlation analysis in integrated single-cell fibroblast-epithelial clusters listed the top 100 correlated genes to TNFRSF12A (Figure 5E, full list at Supplementary Materials Data S4). Most of these genes were tumor cell metastasis-associated genes such as TNC, PLAU, PDPN, SPARC, LUM, and especially SNAI2 (Zinc finger protein SLUG transcription factor). There was a high co-expression of TNFRSF12A and SNAI2 in single-cell data in metastasis conditions (Figure 5F). These two genes were also upregulated together from non- to metastasis conditions. In non-tumor cells (normal cells) of HNSCC samples, no correlation between TNFRSF12A and SNAI2 was found (R = 0.0035); however, a relatively high correlation between TNFRSF12A and SNAI2 was found (R = 0.57) in HNSCC tumor cells (Figure 5G). All above findings indicated the different roles of TNFRSF25 and TNFRSF12A in p-EMT tumor cells in non- vs. metastasis conditions of oral cancer, and TNFRSF12A is highly associated with tumor cell metastasis transcription factor SNAI2.

## 3. Discussion

Current applications for scRNA-seq include the identification of the cell composition of tissues, analysis of the clonal evolution of cancer, understanding the development from stem cells and progenitor cells, cell communication through cell receptor–ligand networks, and cellular responses to genetic manipulation or drugs [16,17]. Our present study analyzed the largest OSCC single-cell RNA-seq data from multi-studies with significant numbers of replicates and metastasis information. By confusing oral locations in non- and metastasis conditions, the analysis has highlighted a complex cellular ecosystem with active communication between CAFs and p-EMT tumor cells.

In the total cell sample of OSCC tumors, we identified 15 main cell types that were confirmed previously in both human and mouse samples [1,5]. The main populations were composed of immune cells and non-immune cells, including cancer-associated cells that were similar to HNSCC data [12,18]. Interestingly, we found a small population of new peripheral helper T cells (Tph) with CXCL13+ and PDCD1/PD-1+ expressions [13,19]. These cells assist B cells within inflamed tissues, including cancer tissues, and are linked to improved survival in patients with malignant tumors [20]. Moreover, the formation of tertiary lymphoid structures within tumors could be supported by CXCL13 produced from Tph cells [21,22], suggesting an important prognostic factor associated with tumor progression and recurrence [23]. These cells could be a candidate immune strategy in OSCC targeted therapies via adaptive antitumor humoral responses in the chronic inflammatory cancer microenvironment.

Aiming at CAFs and p-EMT tumor cells, for the first time, we named the subtypes of these cells within OSCC tumor cells. CAFs were classified as eCAFs with the characteristics of mesenchymal markers, elastic fibers, and ECM proteins and THY1, POSTN, FAP, FN1, and FBLN1. While POSTN (Periostin) associates with epithelial–mesenchymal transitions in cancer and the differentiation of mesenchyme, FAP controls fibroblast growth or epithelial–mesenchymal interactions during development, tissue repair, and epithelial carcinogenesis [15,24]. eCAFs also highly expressed SFRP2 (Secreted frizzled-related protein 2), a soluble modulator of Wnt signaling, which was considered as a potential prognostic marker for uterine cervical preneoplastic lesions progression [25]. We also confirmed the existence of myCAFs (myofibroblastic CAFs), iCAFs (inflammatory CAFs), and apCAFs (antigen-presenting CAFs) in OSCC samples. These cells shared characteristics with previous myCAFs, iCAFs, and apCAFs identified by sc-RNAseq, bulk RNAseq, and functional experiments in pancreatic cancer and breast cancer [14,15,26]. These eCAFs and myCAFs are thought to reside close to the tumor cells and facilitate tumor invasiveness via EMT within TME [11,27]. These cells mediate the immunosuppressive TME by promoting monocyte recruitment and macrophage differentiation (innate immunomodulatory functions) or modulating CD8 T cell function and differentiation (adaptive immune response) [7,28]. iCAFs and apCAFs have formed part of the inflammatory response via chemokine and cytokine production and the capacity to present antigen to CD4+ T cells [15]. The present study has provided new information about the cell types associated with OSCC, especially CAFs’ heterogeneity. Recently, anti-CAF therapy approaches have been developed for cancer immunotherapy; these included FAP depletion, blocking CAF function using inhibitors such as CXCR4 inhibitor and JAK inhibitor, altered CAF activation by vitamin A and D, and ECM-targeted therapy by associated signaling pathways and proteins [15].

In epithelial tumor cells, two subclusters of cycling tumor cells were assigned with high expressions of the cell cycle, proliferation, and DNA repair genes; this indicated OSCC tumor progression in TME. In particular, a population of p-EMT tumor cells was labeled with high expressions of SPRR1B (Small Proline Rich Protein 1B, a cross-linked envelope protein of keratinocytes), a prognostically predictive biomarker for lung adenocarcinoma, overexpressed in human OSCC stem-like cells relating to cell growth through the activation of MAP kinase signals [29,30]. CDH1 (E-cadherin) was found to be downregulated in metastasis p-EMT tumor cells. The downregulation of E-cadherin in OSCC is associated with the delocalization of these proteins from the membrane to the cytoplasm. It was correlated to aggressive, unwell differentiated, high-grade carcinomas, and overall survival probability [31]. Studies have reported the p-EMT programs, subclusters, and how p-EMT predicts nodal metastasis [18]. p-EMT programs boost tumor cells’ invasive potential and hence encourage local invasion in tumor-surrounding tissues, resulting in minimum residual disease, which is a source of recurrence [32]. Here, we have pictured how p-EMT tumor cells interact with the closest cells to the tumors (eCAFs and myCAFs) at the single-cell level. To investigate the interactions between CAFs and epithelial cancer cells, we applied CellPhoneDB. This method stores ligand-receptor interactions and other interacting partners, including their subunit architecture and gene and protein identifiers [33]. Metastasis eCAFs and myCAFs significantly increased NOTCH and Wnt signaling as well as collagen and ECM connection in p-EMT tumor cells. Previously, NOTCH and Wnt signaling indicated important roles in altering cell behaviors, including OSCC cell proliferation, the induction of EMT, and tumor progression, and they were emerging therapeutic targets for clinical cancer treatment [34,35,36,37,38,39]. However, the precise role of NOTCH receptors has yet to be determined. Studies showed that NOTCH2 inhibits myocyte proliferation and NOTCH3 promotes cell proliferation and protects cells from apoptosis [40]. In OSCC, the opposing roles of NOTCH signaling in cancer were observed in which NOTCH receptor family members can be tumor-suppressors (NOTCH1, 2) or oncogene (NOTCH1, 3) [37,41,42]. As a result, medicines that block or promote NOTCH signaling may be particularly useful depending on the situation. For example, NOTCH inhibitors such GSI (Gamma-Secretase Inhibitor) and MK-0752 for solid tumors have been tested in Phase I clinical trials, resulting in stable disease [42,43]. EMT and tumor cells’ invasiveness in various malignancies were also enhanced by collagen-rich CAFs. CAFs can generate gaps in the matrix or basement membrane to guide tumor cells’ collective invasion while remaining connected via cell–cell junctions [44,45]. This is called CAF-mediated TME remodeling, which helps promote tumor invasion and metastasis. Hence, targeting the tumor-collagen interface may be a novel strategy in the treatment of aggressive cancer [8].

We discovered the opposite roles of TNFSF12 (TWEAK) in eCAFs/myCAFs and p-EMT tumor cells in non- vs. metastasis conditions (Figure 5H). Multifunctional cytokine TWEAK controls various cellular activities, including tumor cell proliferation, migration, differentiation, apoptosis, angiogenesis, and inflammation [46]. TWEAK can bind to death receptor 3 (DR3/TNFRSF25) to facilitate apoptosis [47]. Because TWEAK is a proinflammatory cytokine, prolonged TWEAK–Fn14 (TWEAKR/TNFRSF12A) axis signaling could theoretically play a role in diseases induced by excessive or aberrant inflammatory responses in humans, including rheumatoid arthritis and multiple sclerosis. Conversely, TWEAK-Fn14 cytokine-receptor axis activation resulted in increased proliferation, invasion, and the migration of tumor cells and angiogenesis, pro-inflammatory cytokine expression, and EMT [48,49]. TWEAKR was highly expressed in glioma and esophageal adenocarcinoma cell lines and cooperated with invasive activity; in addition, it is a potential cell surface portal for the targeted delivery of glioblastoma therapeutics. Several TWEAK–TWEAKR-targeting therapies for malignant tumors have been developed, such as anti-TWEAK neutralizing mAb, soluble Fn14-Fc decoy protein, and anti-Fn14 mAb with promising outcomes in vitro and in vivo [46,48]. Here, we found that TNFRSF12A correlated to a low HNSCC survival probability; TNFRSF12A also co-expressed with SNAI2, a signature transcription factor in HNSCC. These findings and previous reports suggest that Fn14 (TWEAKR/CD266/TNFRSF12A) could be a potential invasive/metastatic biomarker and therapeutic target in HNSCC and OSCC. The p-EMT program may play a role in the poor prognosis of HNSCC patients. The p-EMT-related genes discovered in this work can be employed as an HNSCC prognostic marker. It is also likely that lymph-node metastasis is a poor approximation for distant metastasis, yet the pEMT algorithm still offers good predictions [11]. From our study, TNFRSF25 and TNFRSF12A could be a good biomarker pair for the prognostic and p-EMT targeting therapies of OSCC and HNSCC. Regarding the limitation of the present study, the results of our analysis of targeted molecules should be confirmed by further functional experiments on cancer cell lines, surgical specimens, and animal models. In the next study, we also need to continuously discover subtypes and interactions between OSCC endothelial tumor cells within the TME at the single-cell level.

## 4. Materials and Methods

### 4.1. Data Obtaining and Preprocessing

Single-cell RNA-seq datasets for oral squamous cell carcinoma (GSE103322 and GSE164690) were downloaded from Gene Expression Omnibus database (https://www.ncbi.nlm.nih.gov/geo/query/acc.cgi?acc=GSE103322, (accessed on 4 May 2022), https://www.ncbi.nlm.nih.gov/geo/query/acc.cgi?acc=GSE164690, (accessed on 4 May 2022)) [12,18]. We combined our single-cell RNA-seq data as previously reported [1]. Only samples with oral cavity site, primary tumor, and metastasis status characteristics were included in the downstream analysis using the Seurat R package (v4) [50]. We primarily filtered genes expressing in less than 3 cells and cells with fewer than 200 genes. Cells with greater than 5% mitochondrial reads and 7000 nFeature_RNA and 18,000 nCount_RNA were also excluded during quality control.

### 4.2. Single-Cell RNA-seq Integration and Computational Methods

In this study, we used the SCTransform method, an improved method for the normalization of scRNA-seq based on regularized negative binomial regression [51,52]. Three thousand selected variable features across the samples were used to scale and normalize the data and repressed mitochondrial genes. The integration method of Seurat v4 includes a set of methods to align shared cell populations across datasets [50]. The first 30 principal components of the integrated data were retained for UMAP projection and clustering analysis based on graph-based clustering approach [53,54,55].

To visualize single cells on a UMAP plot according to gene expression, we used FeaturePlot with minimum and maximum cutoff values of 10 and 90%, respectively. The dotplot function was used to visualize how feature expression changes across different clusters. The dot’s size encodes the percentage of cells within a cluster, while the color encodes the AverageExpression level across all cells within a cluster. Wilcoxon Rank Sum test, with an adjusted *p*-Value < 0.05 by Bonferroni correction, was used to identify the markers of each cluster or each condition. Pseudo-bulk RNA-seq analysis calculated the average gene expression and differentially expressed genes across different conditions by using the AverageExpression function.

We applied the Pearson method to calculate the correlation expression of all features with an interested gene in integrated data. Then, the correlation network of the top correlated genes was visualized by visNetwork package (v2) (https://cran.r-project.org/web/packages/visNetwork/index.html (accessed on 4 May 2022)). The FeaturePlot function with a blending mode was used to verify the co-expression of 2 genes.

### 4.3. Enrichment and Cell–Cell Communication Analysis

We applied Metascape (v3.5) (https://metascape.org (accessed on 4 May 2022)) for enrichment clustering and multi-gene-list meta-analysis based on terms across different ontology sources. We then identified all statistically enriched terms, accumulative hypergeometric p-Values, and enrichment factors for each gene set [56]. Cell–cell communication analysis was performed using available receptors, ligands, and their interactions from the single-cell transcriptomics data by Python package CellPhoneDB (v3) (https://www.cellphonedb.org, (accessed on 4 May 2022)). This method utilizes information from the UniProt, Ensembl, PDB, the IMEx consortium, and IUPHAR databases. Both ligands and receptors, representing heteromeric complexes accurately form the subunit’s architecture [33].

### 4.4. Survival Analysis, Correlation Analysis and Images of Protein Expression

Survival analysis was performed based on gene expression levels (TNFRSF12A vs. TNFRSF25) using head–neck squamous carcinoma in The Cancer Genome Atlas (TCGA) database (https://portal.gdc.cancer.gov, (accessed on 4 May 2022)) in by GEPIA (v2) [57,58]. The method applied a log-rank test for the hypothesis evaluation. We included the Cox proportional hazard ratio and the 95% confidence interval in the survival plot. Pairwise gene correlation analysis for TNFRSF12A and SNAI2 in the TCGA database (normal and HNSCC tumor tissues) was performed using Pearson correlation statistics.

The protein expression images of immunohistochemistry staining of TWEAK receptor (TWEAKR/CD266/TNFRSF12A) using antibody HPA007853 in HNSCC primary tumors were obtained from Human Protein Atlas v.21 (www.proteinatlas.org/ENSG00000006327-TNFRSF12A/pathology/head + and + neck + cancer, (accessed on 4 May 2022)) [59]. The expressions of TWEAKR were classified as low, medium, and high levels in tumors.

## 5. Conclusions

In conclusion, we have completed the picture of CAFs and p-EMT tumor cells in OSCC TME at the single-cell level. The analysis discovered four subtypes of CAFs, one p-EMT tumor cell and cycling tumor cell population, as well as the roles of TNFSF12–TNFRSF25/TNFRSF12A interactions in CAFs and p-EMT tumor cells during tumor metastasis at the single-cell level. Our study suggests the prediction of therapeutically targetable checkpoint receptor–ligand interactions between CAFs and p-EMT tumor cells in OSCC regarding the metastasis status.

## Data Availability

Not applicable.

## Supplementary Materials

The following supporting information can be downloaded at: https://www.mdpi.com/article/10.3390/ijms23126470/s1.
